# B7H3与纤维连接蛋白相互作用对人慢性髓性白血病细胞凋亡影响的初步探讨

**DOI:** 10.3760/cma.j.issn.0253-2727.2021.11.009

**Published:** 2021-11

**Authors:** 美云 孙, 金晶 谢, 东泽 张, 光波 张

**Affiliations:** 1 苏州大学医学部 216007 Department of Medicine, Soochow University, Suzhou 216007, China; 2 江苏省临床免疫学重点实验室，苏州 216007 Jiangsu Key Laboratory of Clinical Immunology, Suzhou 216007, China; 3 苏州大学附属第一医院 216007 The First Affiliated Hospital of Soochow University, Suzhou 216007, China

**Keywords:** K562细胞, 细胞黏附, 细胞凋亡, B7H3, 纤维连接蛋白, K562 cells, Cell adhesion, Cell apoptosis, B7H3, Fibronectin

## Abstract

**目的:**

探讨B7H3和纤维连接蛋白（FN）相互作用对人慢性髓性白血病细胞系K562细胞凋亡的影响。

**方法:**

采用流式细胞术检测K562细胞中B7H3分子的表达，构建B7H3过表达细胞。采用免疫共沉淀技术检测B7H3与FN的相互作用。添加外源FN后，通过细胞实验检测细胞黏附和细胞凋亡的变化。Western blot法检测凋亡相关蛋白及PI3K/AKT信号通路的变化。

**结果:**

① K562细胞低表达B7H3分子，慢病毒转染后得到稳定表达B7H3的细胞系K562 OE-B7H3及其对照细胞系K562 NC-B7H3细胞。②B7H3与FN之间存在相互作用（*P*＝0.036）。③B7H3与FN的相互作用促进细胞黏附（*P*<0.05），抑制细胞凋亡（*P*<0.05）。④B7H3与FN相互作用激活PI3K/AKT信号通路（*P*<0.05）。

**结论:**

B7H3与FN相互作用促进了细胞黏附，可能通过激活PI3K/AKT信号通路抑制K562细胞的凋亡。

## Abstract (en)

**Objective:**

To explore the effect of the interaction between B7H3 and fibronectin (FN) on the apoptosis of human chronic myeloid leukemia K562 cells.

**Methods:**

The expression of B7H3 molecules in K562 cells was detected using flow cytometry and B7H3 overexpressing cells were constructed. The interaction between B7H3 and FN was detected using the co-immunoprecipitation technology. After adding exogenous FN, cell experiments were performed to detect changes in adhesion and cell apoptosis. The changes in apoptosis-related proteins and PI3K/AKT signaling pathway were detected using Western blot.

**Results:**

The expression of B7H3 was low in K562, and the cell line K562 OE (overexpression)-B7H3 and the control cell line K562 NC (negative control)-B7H3 were obtained after lentivirus transfection. There is an interaction between B7H3 and FN (*P*＝0.036), and this interaction promoted cell adhesion (*P*<0.05), inhibited cell apoptosis (*P*<0.05), and activated the PI3K/AKT signaling pathway (*P*<0.05).

**Conclusion:**

B7H3 interacts with FN to promote cell adhesion and may inhibit K562 cell apoptosis by activating the PI3K/AKT signaling pathway.

慢性髓性白血病（CML）属于骨髓增生性疾病，约占成人白血病的15％[1]。2000年伊马替尼问世，CML的年病死率降至1％～2％[2]。B7H3在发现时被认为是T细胞的刺激蛋白，但是目前的诸多研究显示B7H3作为T细胞的抑制剂促进肿瘤的侵袭和增殖[3]。研究表明，双特异性的B7H3抗体处理K562细胞18 h后，对细胞表现出强的杀伤能力，而用抗B7H3单抗阻断后，杀伤能力减弱，进一步提示B7H3可以作为血液病免疫疗法的新靶点[4]。已有研究表明B7H3与细胞之间及细胞与细胞外基质之间的黏附相关[5]，而纤维连接蛋白（Fibronectin, FN）的基本作用就是促进细胞黏附[6]。有学者采用FN多克隆抗体检测到FN和B7H3的表达，于是我们猜测FN与B7H3分子可能通过形成天然复合物发挥作用。本研究中我们对B7H3与FN相互作用对CML细胞凋亡的影响及其作用机制进行初步研究。

## 材料与方法

一、材料

人慢性髓性白血病细胞系K562细胞购自中国科学院细胞库；RPMI 1640培养基、胎牛血清为美国BI公司产品；PBS为美国 Hyclone公司产品；FN、puromycin为北京索莱宝科技有限公司产品；IP裂解液、RIPA Lysis 缓冲液、SDS-PAGE蛋白质上样缓冲液、100×青霉素和链霉菌混合液、PMSF、β-Actin、IgG、Protein A+G Agrose购自上海碧云天生物技术有限公司；Annexin Ⅴ-PE/7-AAD凋亡检测试剂盒为美国BD公司产品；转录试剂盒为诺唯赞公司产品；Caspase-3、Caspase-9、Bax、AKT、p-AKT、B7H3、p53、FN抗体为美国Proteintech公司产品；Caspase-8、PI3K、p-PI3K抗体为美国Abcam公司产品；Bcl-2抗体为美国CST公司产品；病毒为上海吉玛基因公司产品；GAPDH、B7H3引物购自金唯智生物科技有限公司。

二、方法

1. 细胞培养：K562细胞用含10％热灭活胎牛血清的RPMI 1640培养基，37 °C、5％ CO_2_的潮湿培养箱中培养。

2. 流式细胞术检测K562细胞中B7H3的表达：离心收集细胞，每管加入10^5^个细胞后，加入1 µl PE标记的鼠抗人B7H3抗体，避光4 °C孵育30 min，洗涤，上机检测。

3. 慢病毒转染K562细胞：将K562细胞以2×10^5^细胞/ml接种于6孔板中，按照慢病毒转染说明书进行操作。转染72 h后加入含2 µg/ml puromycin的RPMI 1640培养基，经筛选分别得到稳定表达B7H3的K562-OE细胞株以及对照K562-NC细胞株，并对两组细胞进行检测和鉴定。

4. 实时定量PCR检测B7H3 mRNA的表达：将已筛选两周的K562-OE和K562-NC细胞用RNA提取试剂盒提取总RNA。使用日本TaKaRa公司试剂盒、10 µl体系进行逆转录，实时定量PCR测定B7H3在基因水平上的表达，B7H3相对表达量采用2^−∆∆Ct^法进行计算。

5. 免疫共沉淀：收集K562细胞，取细胞沉淀加入1 ml IP裂解液，冰上裂解5 min，4 °C 12 000×*g*离心，收集上清。按IgG和Protein A+G Agarose说明书操作。经洗涤后向上清液中加入1×蛋白上样缓冲液，金属浴100 °C煮5 min，取全部样品用于Western blot检测。

6. 细胞黏附实验：20 µg/ml FN包被于96孔板中，并以PBS作为阴性对照，2％ BSA封闭1 h。封闭结束后，用无血清培养基洗涤96孔板。收集对数生长期的ctrl-NC、FN-NC、ctrl-OE、FN-OE K562细胞，将细胞重悬于含1％FBS的培养基中，制成5×10^2^细胞/µl悬液，每孔添加100 µl，每组设5个复孔，在细胞培养箱中培养1 h。PBS洗涤，经多聚甲醛固定和结晶紫染色后，冲洗并干燥，在显微镜下观察黏附细胞的数量并拍照，选取其中3张照片用Image J软件计数统计。

7. 流式细胞术检测细胞凋亡：将K562细胞接种于6孔板，当细胞处于对数生长期时，对细胞进行如下分组：饥饿+ctrl-NC（无血清RPMI 1640培养NC细胞），饥饿+FN-NC（无血清RPMI 1640和外源FN培养NC细胞），饥饿+ctrl-OE（无血清RPMI 1640培养OE细胞），饥饿+FN-OE（无血清RPMI 1640和外源FN培养OE细胞），培养24 h。收集细胞培养上清和细胞，预冷的PBS洗涤两次后，用100 µl 1×Binding 缓冲液重悬细胞，分别加入5 µl Annexin Ⅴ-PE抗体和7-AAD抗体，室温避光孵育15 min，加入300 µl 1×Binding 缓冲液，于1 h内上机检测。

8. Western blot法检测凋亡相关蛋白与PI3K/AKT信号通路相关分子的表达：收集经饥饿处理的四组K562细胞，提取蛋白并用Western blot法检测B7H3、Caspase-9、Caspase-8、Apaf-1、Cleaved PARP、Cytochrome C、Caspase-3、Bax、Bcl-2、p53、p-PI3K、PI3K、p-AKT、AKT蛋白水平的表达变化。

三、统计学处理

实验数据用GraphPad Prism 7.0、Image J以及FlowJo 7.6分析且所有实验均重复3次，两组之间的比较应用*t*检验，*P*<0.05为差异有统计学意义。

## 结果

1. B7H3 OE-K562细胞株的构建：应用流式细胞术检测B7H3的表达，NC组B7H3的表达率为15.7％，OE组B7H3的表达率为69.6％（图1A），表明B7H3的转染效率较高。Western blot法检测两组细胞B7H3在蛋白水平上的表达，OE组B7H3的表达水平明显高于NC组，差异有统计学意义（*P*＝0.003）（图1B）。实时定量PCR检测B7H3在mRNA水平上的表达，NC组B7H3的表达水平明显低于OE组，且差异有统计学意义（*P*＝0.009）（图1C）。

**图1:**
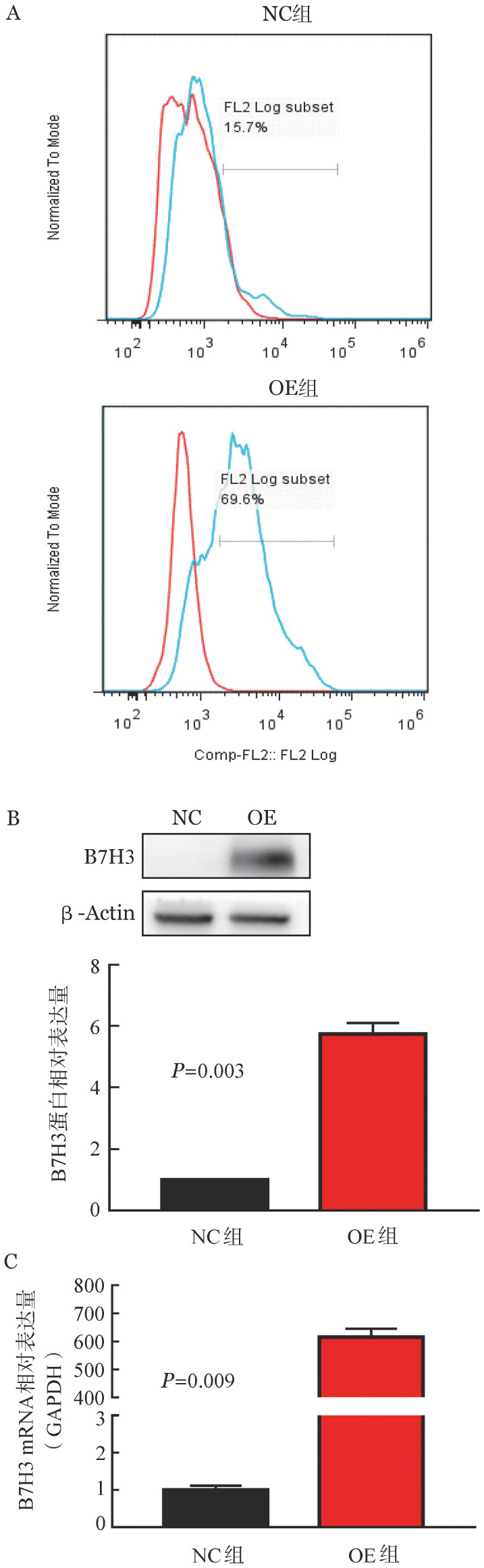
过表达B7H3 K562细胞株的构建与鉴定（实验重复3次） A：流式细胞术检测对照（NC）组和过表达（OE）组细胞B7H3的表达；B：Western blot法检测B7H3在蛋白水平的表达；C：实时定量PCR检测B7H3在基因水平的表达

2. B7H3与FN的相互作用：如图2A所示，我们从TCGA数据库中查询到，B7H3与FN在白血病细胞中存在一定的相关性（*P*＝0.036）。随后，我们通过免疫共沉淀实验验证B7H3与FN在K562细胞中是否具有相互作用。结果如图2B所示，FN在K562细胞中被B7H3所沉淀。以上结果表明B7H3与FN存在相互作用。

**图2:**
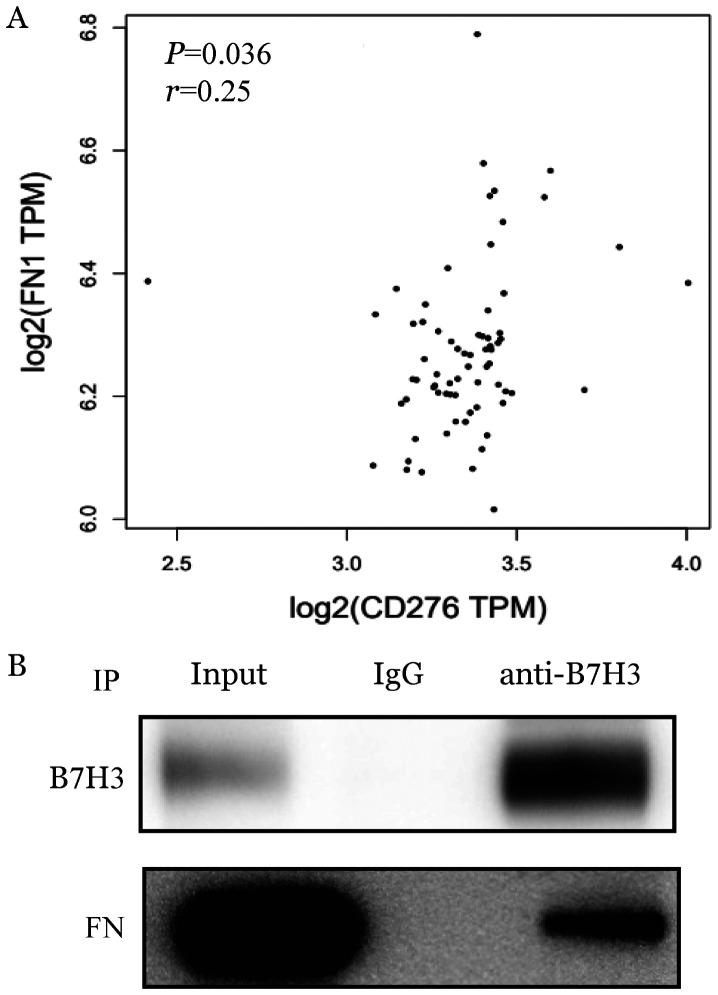
B7H3与纤维连接蛋白（FN）相互作用及其调节关系的检测 A：慢性髓性白血病患者中B7H3与FN表达的相关性分析（数据源自TCGA数据库）；B：免疫共沉淀法检测B7H3与FN之间的相互作用

3. B7H3与FN的相互作用对细胞黏附的影响：如图3所示，与ctrl-NC组细胞相比，ctrl-OE组细胞的黏附率显著上升（*P*＝0.022）；ctrl-NC组与ctrl-OE组细胞在分别用外源FN刺激后，细胞黏附率均显著上升（*P*＝0.016和*P*＝0.022），且ctrl-NC组细胞在外源FN刺激后黏附率上升幅度明显低于ctrl-OE组细胞在外源FN刺激后黏附率上升幅度。表明B7H3与FN的相互作用可以进一步促进细胞黏附。

**图3:**
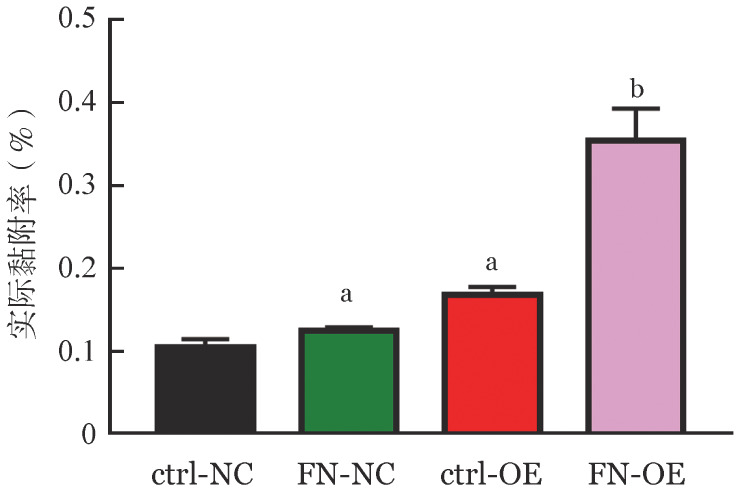
B7H3与纤维连接蛋白（FN）相互作用对细胞黏附的影响（实验重复3次） ctrl-NC组：对照细胞；FN-NC：外源FN培养对照细胞；ctrl-OE组：B7H3过表达细胞；FN-OE：外源FN培养B7H3过表达细胞。^a^与ctrl-NC组比较，^a^*P*<0.05；与ctrl-OE组比较，^b^*P*<0.05

4. B7H3与FN相互作用对K562细胞凋亡的影响：如图4所示，与ctrl-NC组相比，FN-NC组细胞的早期或晚期细胞凋亡均无显著变化（*P*>0.05）；而FN-OE组与ctrl-OE组相比，细胞的早期凋亡率下降（*P*＝0.018），晚期细胞凋亡率差异无统计学意义（*P*＝0.176）。随后，我们又检测了凋亡分子在蛋白和mRNA水平的表达变化。如图5A和5B所示，ctrl-NC组细胞在添加FN后，Cleaved PARP、Cytochrome C、Apaf-1、Caspase-8和Caspase-9的表达差异均无统计学意义（*P*值均>0.05）；但ctrl-OE组细胞加入FN后，Cleaved-PARP、Cytochrome C、Apaf-1、Caspase-8和Caspase-9的表达均降低（*P*值均<0.05）。表明B7H3与FN的相互作用抑制K562细胞凋亡。

**图4:**
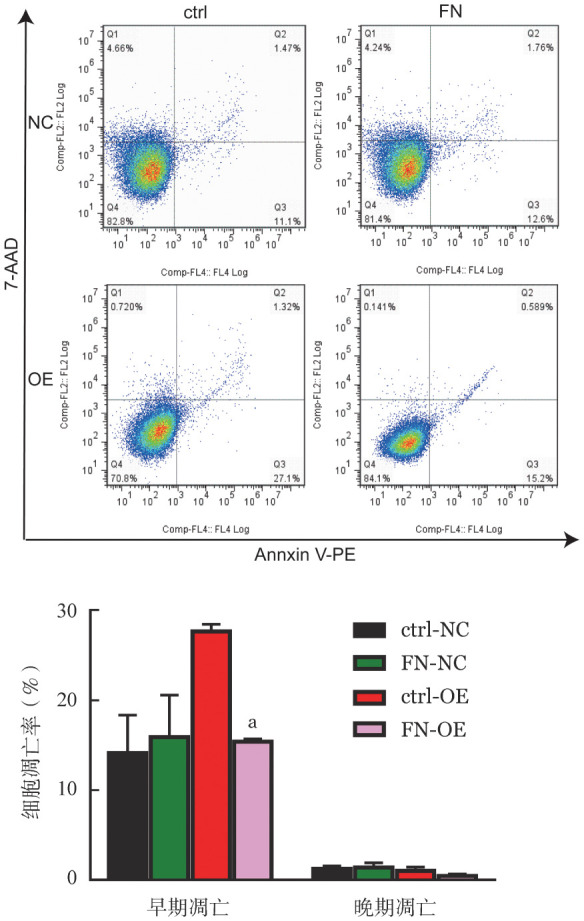
流式细胞术检测B7H3与纤维连接蛋白（FN）相互作用对细胞凋的影响（实验重复3次） ctrl：无血清RPMI 1640培养；FN：无血清RPMI 1640和外源FN培养；NC：对照细胞；OE：B7H3过表达细胞。与ctrl-NC组比较，*P*值均>0.05；与ctrl-OE组比较，^a^ *P*<0.05

**图5:**
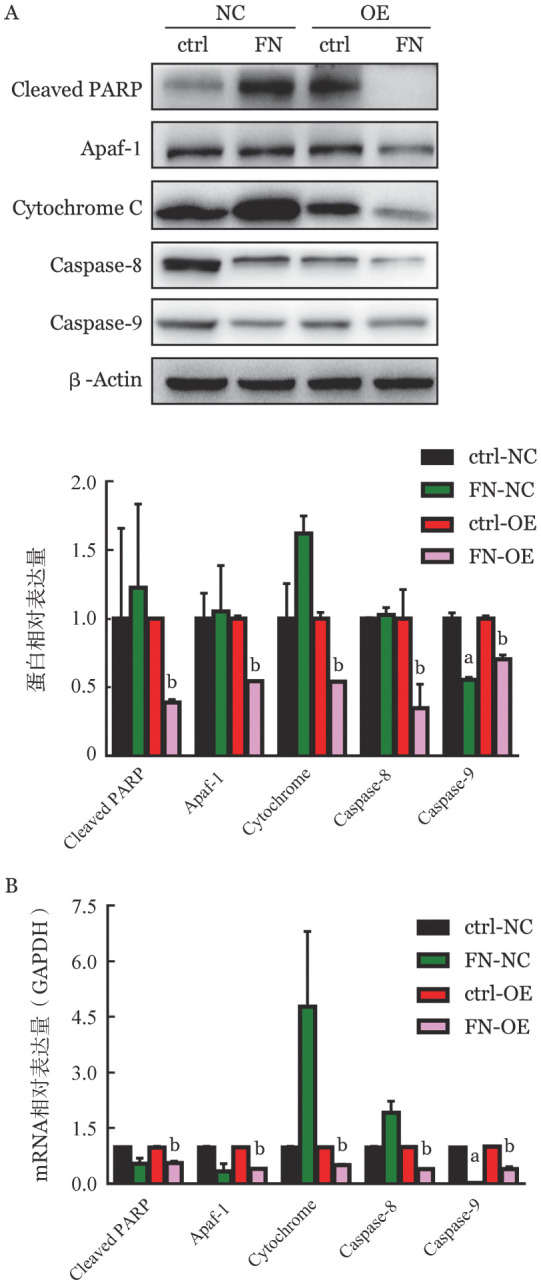
Western blot法（A）和实时定量PCR法（B）检测B7H3与纤维连接蛋白（FN）相互作用对细胞凋亡相关分子表达的影响（实验重复3次） ctrl：无血清RPMI 1640培养；FN：无血清RPMI 1640和外源FN培养；NC：对照细胞；OE：B7H3过表达细胞。与ctrl-NC组比较，^a^ *P*<0.05；与ctrl-OE组比较，^b^ *P*<0.05

5. B7H3与FN的相互作用激活PI3K/AKT信号通路：为了进一步探讨B7H3与FN相互作用抑制K562细胞凋亡的机制，我们检测了PI3K/AKT信号通路相关分子在蛋白和mRNA水平的表达。如图6所示，ctrl-OE组在添加FN后，p-PI3K、p-AKT和Bcl-2的表达均上调（*P*值均<0.05），并且p53、Bax和Caspase-3表达均下调（*P*值均<0.05），但总的PI3K和AKT表达保持不变（*P*>0.05）。表明B7H3与FN的相互作用是通过激活PI3K/AKT信号通路来抑制K562细胞的凋亡。

**图6:**
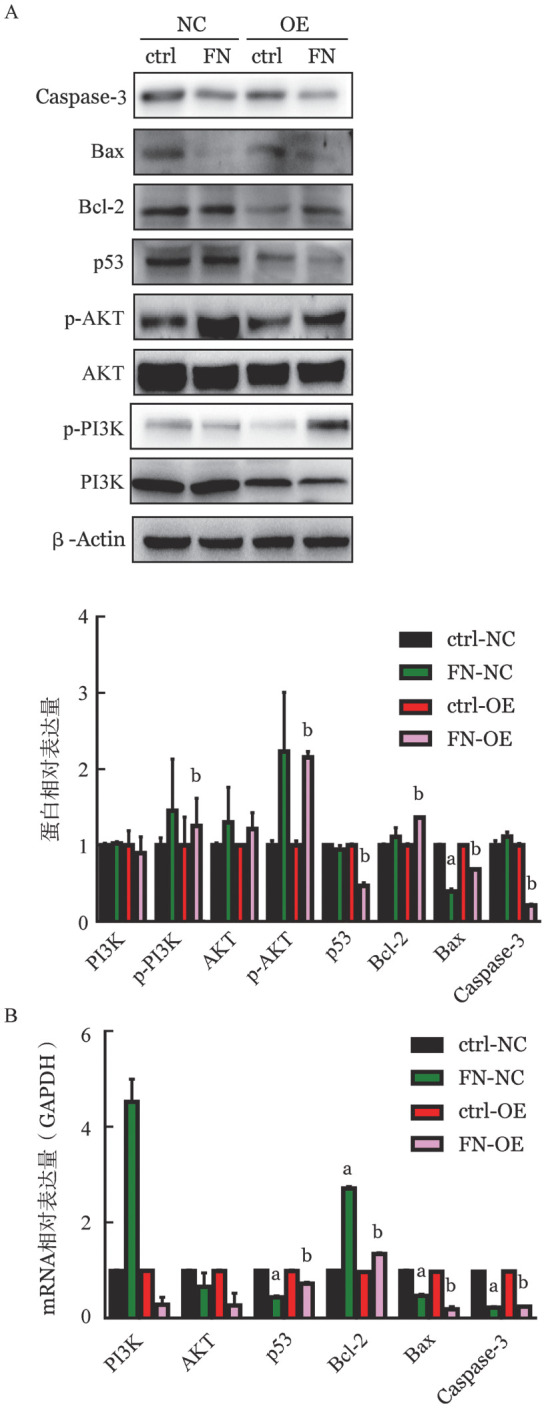
Western blot法（A）和实时定量PCR法（B）检测B7H3与FN相互作用对PI3K/AKT信号通路相关分子表达的影响（实验重复3次） ctrl：无血清RPMI 1640培养；FN：无血清RPMI 1640和外源FN培养；NC：对照细胞；OE：B7H3过表达细胞。与ctrl-NC组比较，^a^ *P*<0.05；与ctrl-OE组比较，^b^ *P*<0.05

## 讨论

染色体易位产生的BCR-ABL融合蛋白因其具有酪氨酸激酶活性，可调控细胞内的多条信号通路，致使骨髓细胞恶性增殖，最终引发CML[7]。它的特点是不成熟的白细胞在骨髓内大量聚集，抑制了骨髓的正常造血功能，这些不成熟的白细胞通过血液循环扩散到全身，致使患者出现贫血、脾大、易出血等症状[8]。已有文献报道，降低B7H3的表达后，细胞的黏附能力也随之下降，表明B7H3分子可能通过阻止肿瘤细胞黏附分子与其配体之间的结合来预防癌症转移[9]–[11]。在本实验中，我们用慢病毒升高K562细胞中B7H3的表达，并通过免疫共沉淀技术证实B7H3与FN之间确实存在相互结合并且两者结合之后可以促进细胞黏附。而且，B7H3与FN相互作用后显著抑制细胞凋亡，经实验验证，加入外源FN后，促凋亡蛋白Caspase-8、Caspase-9、Apaf-1、Cleaved PARP和Cytochrome C的表达下降。细胞黏附是肿瘤进展的关键介质，细胞黏附受体会与信号通路相连接，进而调控包括增殖、存活、迁移和分化等在内细胞表型[12]。Caspase-8是黏附细胞死亡的关键介质[13]。在凋亡信号的刺激下，线粒体通透性开放，跨膜电位分解，呼吸链解偶联，增加线粒体基质的渗透压，细胞色素C释放到细胞基质中。当存在ATP时，细胞色素C会与细胞凋亡蛋白激活因子Apaf-1结合形成复合物，该复合物激活Caspase-9，从而激活Caspase-3并切割底物诱导细胞凋亡[14]。Bcl-2通过阻止促凋亡分子从线粒体进入细胞质来阻止细胞凋亡，而Bax通过诱导线粒体外膜的通透性来促进细胞凋亡[15]–[17]。Bax/Bcl-2的比值决定了细胞对死亡刺激的敏感性[18]。Apaf-1在线粒体凋亡途径中起着重要作用，因为它促进了包括形成具有Cytochrome C和dATP的凋亡小体在内的多种加工过程，凋亡小体又可以激活Caspase-3并诱导细胞凋亡[19]。越来越多的证据表明，细胞外基质黏附激活了PI3K/AKT信号通路，从而抑制了多种形式的肿瘤细胞死亡[20]–[21]。PI3K是黏附形成的关键调节剂，它可以直接与E-Cadherin结合，从而调节黏附介导的细胞周期进展[22]–[23]。也有研究表明，AKT蛋白的表达下调能够显著诱导细胞凋亡和抑制细胞增殖[24]。目前已经确定AKT信号通路的下游靶蛋白是与细胞凋亡相关的蛋白分子如Bax、Bcl-2和Caspase-3等[25]。活化的AKT可能会通过p53途径和Bcl-2蛋白家族影响细胞周期进程和肿瘤生长[26]。在本实验中，B7H3与FN相互作用之后，p-PI3K、p-AKT和Bcl-2表达上调，p53、Bax和Caspase-3表达下调，但是总的PI3K和AKT的表达没有变化即B7H3与FN相互作用可能通过激活PI3K/AKT信号通路进而抑制K562细胞凋亡。

综上所述，B7H3与FN相互作用促进细胞黏附，抑制K562细胞凋亡，并且可能在将来成为治疗CML的潜在靶标，但是其更加深入的调节机制有待我们进一步验证。本实验的局限性主要为缺少动物实验和临床数据的验证，我们会以此为基础，进行下一步研究。
